# A Case of C3 Nephritis With a Rare Variant of the CFHR5 Gene

**DOI:** 10.7759/cureus.55102

**Published:** 2024-02-27

**Authors:** Hisashi Kamido, Shinya Yamamoto, Hideki Yokoi, Masashi Mizuno, Motoko Yanagita

**Affiliations:** 1 Nephrology, Graduate School of Medicine, Kyoto University, Kyoto, JPN; 2 Nephrology, Nagoya University, Nagoya, JPN; 3 Human Biology, World Premier International Research Center Initiative-Institute for the Advanced Study of Human Biology (WPI-ASHBi), Kyoto, JPN

**Keywords:** cfhr5, p453s, complement factor h related protein, c3 nephritis, c3 nephropathy

## Abstract

C3 nephropathy is a renal disease caused by the aberrant activation of the alternative complement pathway. The long-term renal prognosis of C3 nephropathy is generally poor, and elucidation of its pathogenesis is clinically important. Genetic abnormalities within complement genes, encompassing autoantibodies targeting complement components and complement factor H-related proteins (CFHRs), can lead to abnormal complement activation. *CFHR5* is one of the best-known responsible genes for C3 nephritis. Moreover, the renal prognosis can vary depending on the specific type of genetic mutation. Here, we report the case of a young woman with C3 nephritis and a heterozygous rare variant, P453S, in *CFHR5*. The P453S variant, characterized by amino acid substitutions with a low allele frequency, was located in the region essential for CFHR5 protein function, and multiple in silico analyses were done suggesting the pathological significance of P453S. The renal function of our patient remains stable. The P453S variant might contribute to the suppression of the CFHR5 protein’s function, resulting in gradual complement progression and a favorable renal prognosis.

## Introduction

C3 nephropathy is a renal disease caused by the aberrant activation of the alternative complement pathway, leading to the deposition of C3 and its degradation products in the glomerulus and subsequent progressive renal injury. This disease is classified into two categories: dense deposit disease (DDD) and C3 nephritis. DDD is characterized by electron-dense deposits in the glomerular basement membrane (GBM), whereas C3 nephritis is characterized by deposits along the subendothelial side of the mesangium and GBM [1].

The long-term renal prognosis of C3 nephropathy is generally poor, and elucidation of its pathogenesis is clinically important [2]. Recent studies have demonstrated that genetic abnormalities in complement genes, including C3, complement factor H (CFH), complement factor I (CFI), complement factor B (CFB), complement factor H-related protein (CFHR), CD46, and autoantibodies targeting complement components such as C3Nef and anti-H antibodies, can cause abnormal complement activation [3,4]. Complement gene abnormalities and autoantibodies account for 24% of patients with C3 nephritis and 35% with DDD [5]. *CFHR5* has been one of the best-known genes responsible since an autosomal-dominant family of C3 nephritis was reported in 2010 [4,6]. Here, we report the case of a young woman with C3 nephritis and a heterozygous rare variant, P453S, in *CFHR5*.

## Case presentation

A 23-year-old woman with no family history of renal disease presented to our nephrology department with a complaint of proteinuria and occult blood in urine during a routine medical checkup. The patient was asymptomatic with no evidence of renal function deterioration, but hematuria was frequently noted during her school examinations eight years ago, and both hematuria and proteinuria became evident three years ago.

On admission, the patient's height was 158 cm, weight was 54 kg, and body mass index was 21.5 kg/m^2^. Her blood pressure was 130/77 mmHg and heart rate was 68 bpm. A physical examination revealed no remarkable findings. Laboratory tests showed creatinine 0.56 mg/dL, C3 19.2 mg/dL, C4 25.2 mg/dL, CH50 < 14 U/mL (Table 1). Autoantibodies, monoclonal proteins, and hepatitis virus infection were not detected. Urine analysis revealed dysmorphic hematuria and a urine protein creatinine ratio of 0.53 g/gCr. Serum level of Ba was 700.1 ng/mL (reference range (RR): 419.6-1714.0 ng/mL), plasma level of Ba was 645.8 ng/mL (RR: 275.6-685.2 ng/mL), serum level of C5a was 17.0 ng/mL (RR: 0.50-32.33 ng/mL), plasma level of C5a was 12.6 ng/mL (RR: 0.20-15.62 ng/mL), serum level of sC5b-9 was 1413.4 ng/mL (RR: 148.0-1243.6 ng/mL), plasma level of sC5b-9 was 2282.9 ng/mL (RR: 37.0-260.6 ng/mL), and anti-H antibodies were negative (Table 1).

**Table 1:** Laboratory Data MPO: myeloperoxidase; ANCA: anti-neutrophil cytoplasmic antibody; dsDNA: double-stranded deoxyribonucleic acid; GBM: glomerular basement membrane; PR3: proteinase 3

Laboratory tests	Results	Normal range	Laboratory tests	Results	Normal range
Urinalysis					
Protein	2+	-	Protein creatinine ratio (g/gCr)	0.53	<0.15
Occult blood	2+	-	β2-microglobulin (mg/L)	0.055	<0.2
Dysmorphic erythrocytes (/HPF)	5-9	<5	N-acetyl-β-D-glucosaminidase (U/L)	1.5	<11.5
Leukocytes (/HPF)	5-9	<5			
Blood tests					
Cystatin C (mg/dL)	0.63	0.61-1.05	Immunoglobulin G (mg/dL)	1563	870-1700
White blood cells ( x 10^3^/μL)	8.36	4-10	Immunoglobulin A (mg/dL)	256	110-410
Hemoglobin (g/dL)	12.4	12-15	Immunoglobulin M (mg/dL)	182	33-190
Platelet ( x 10^3^/μL)	265	150-450	Serum C3 (mg/dL)	19.2	80-140
Aspartate aminotransferase (IU/L)	18	10-35	Serum C4 (mg/dL)	25.2	11-34
Alanine aminotransferase (IU/L)	11	10-35	CH50 (/mL)	<14	30-45
Lactate dehydrogenase (IU/L)	155	120-245	Antistreptolysin-O (IU/mL)	66	<250
Total protein (g/dL)	7.3	5.5-8.0	Antinuclear antibody	<40	<40
Albumin (g/dL)	4.2	3.5-5.5	Anti-dsDNA antibodies (IU/mL)	1.3	<10
Urea nitrogen (mg/dL)	12	6-20	MPO-ANCA (U/mL)	<1.0	<3.5
Creatinine (mg/dL)	0.56	0.50-0.90	PR3-ANCA (U/mL)	<1.0	<2.0
Sodium (mEq/L)	141	133-145	Anti-GBM antibody (U/mL)	<2.0	<7
Chloride (mEq/L)	105	95-108	Hepatitis B surface antigen	-	-
Calcium (mg/dL)	9.3	8.8-10.4	Anti-hepatitis C virus antibody	-	-
C reactive protein (mg/dL)	0.1	<0.3	Cryoglobulin	-	-
Plasma Ba (ng/mL)	645.8	275.6 - 685.2	M protein	-	-
Plasma C5a (ng/mL)	12.6	0.20 - 15.62	Serum Ba (ng/mL)	700.1	419.6-1714.0
Plasma sC5b-9 (ng/mL)	2282.9	37.0 - 260.6	Serum C5a (ng/mL)	17	0.50-32.33
Anti-H antibodies	-	-	Serum sC5b-9 (ng/mL)	1413.4	148.0-1243.6

**Figure 1:**
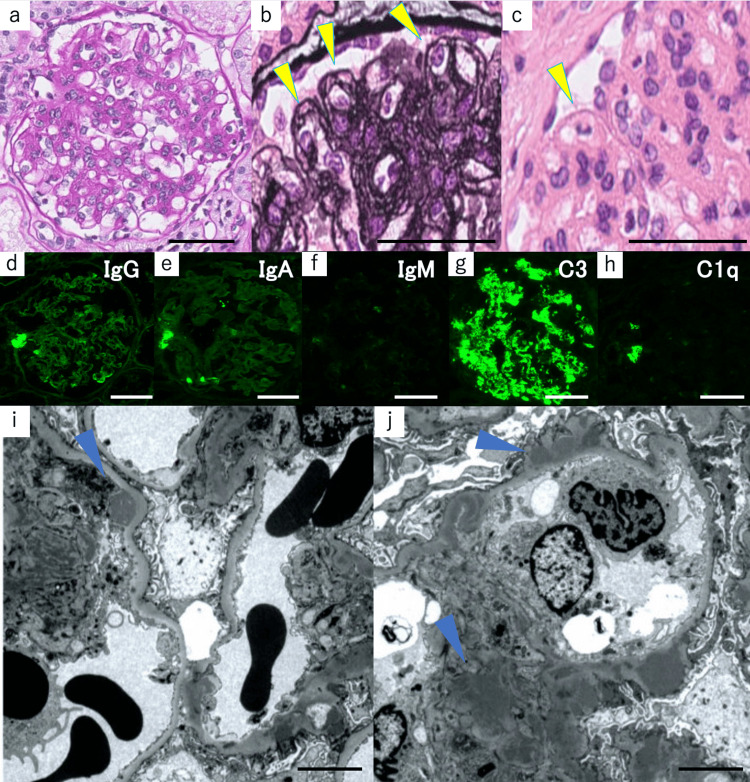
Renal Histological Findings Light microscopy revealed (a) moderate-to-severe mesangial cell proliferation, increased mesangial matrix (periodic acid-schiff stain), (b) double contour, mesangial interposition (periodic acid-methenamine silver stain), and (c) mild lymphocyte and neutrophil proliferation in the capillaries (hematoxylin and eosin stain, yellow arrowhead); (d-h) Immunofluorescence revealed strongly positive for C3 deposition alone, with granular deposition in the mesangium and the capillary; (i-j) Electron microscopy showed significant deposits in the subendothelial (i, blue arrowhead), mesangium, subepithelial and basement membrane (j, blue arrowhead). (a-h) Bars=50μm, (i-j) Bars=5μm.

Renal biopsy revealed 14 glomeruli with no evidence of total or segmental sclerosis or crescent formation. All glomeruli showed moderate-to-severe mesangial cell proliferation and increased mesangial matrix (Figure 1a-1c). Some glomeruli showed double contour, mesangial interposition (Figure 1b), and mild lymphocyte and neutrophil infiltration in the capillaries (Figure 1c). The interstitial fibrosis was mild, and inflammatory cell infiltration and tubular atrophy were not observed. Based on the above results, the light microscopic findings were consistent with membranous proliferative glomerulonephritis (MPGN). MPGN can occur secondary to systemic diseases, including infection-related nephritis, autoimmune diseases, malignancies, genetic diseases, paraproteins, and thrombotic microangiopathies. However, the patient’s symptoms and laboratory findings revealed no systemic signs of these diseases. The fluorescent antibody assay was strongly positive for C3 deposition alone, with granular deposition in the mesangium and capillaries (Figure 1d-1h). Electron microscopy showed significant deposits in the mesangial area, subepithelium, and basement membrane (Figure 1i-1j). Based on these findings, the patient was diagnosed with C3 nephritis.

In addition, genetic analysis of the patient's C3, CFH, CFI, CFB, CFHR, and CD46 coding regions was performed using a next-generation sequencer. The analysis revealed a heterogeneous amino acid substitution variant, c.1357C>T (p. P453S), in the *CFHR5* gene. The variant exhibits an overall allele frequency of 0.00007427, along with specific frequencies of 0.001053 in East Asian populations as reported in Genome Aggregation Database version 2.1.1 [7]. Furthermore, the frequency of the variant is 0.0021 among Japanese populations, according to the Human Genetic Variation Database [8].

Multiple in silico analyses (SIFT, SIFT4G, Polyphen2_HDIV, Polyphen2_HVAR, Mutation Taster, Mutation Assessor, FATHMM, PROVEAN, M_CAP, and CADD) suggested pathological significance (Table 2). Over three years without medical intervention, the patient has consistently exhibited proteinuria levels ranging between 1.0 and 2.0 g/gCr, while her renal function has maintained stability since admission. Following the administration of losartan, there was a reduction in urinary protein excretion to 0.8 g/gCr.

**Table 2:** in Silico Analyses

Analyses	Score	Prediction
SIFT	0.033	Damaging
SIFT4G	0	Damaging
Polyphen2_HDIV	0.997	Probably damaging
Polyphen2_HVAR	0.989	Probably damaging
MutationTaster	0.94211	Polymorphism
Mutation Assessor	2.97	Medium
FATHMM	-0.23	Tolerated
PROVEAN	-7.21	Damaging
M_CAP	0.17	Possibly pathogenic
CADD_phred_hg19	24	Probably damaging

## Discussion

We present the case of a young woman with C3 nephritis and a rare heterozygous variant, P453S, in *CFHR5*. To the best of our knowledge, this is the first report of this variant in patients with C3 nephritis.

The patient had clinical features consistent with Cluster 1 of the previously reported cluster classification of membranoproliferative glomerulonephritis, characterized by a young age of onset, low C3, high C5b-9, absence of crescent on renal biopsy, high mesangial deposits on electron microscopy, and likely pathogenic variants (LPVs) of complement genes, as well as a favorable renal prognosis [9]. However, LPVs in *CFHR5* have yet to be previously reported in Cluster 1, which is known to be abundant in C3 and complement factor B genes [9].

*CFHR5* is a member of the CFH gene cluster on chromosome 1. The CFHR5 protein possesses nine short consensus repeats (SCRs), which is a characteristic feature of complement activation regulators. Notably, SCR8 exhibits C3b-binding properties, analogous to SCR19 of factor H [10]. While the classical and lectin pathways are initiated in response to triggers like infections, the alternative pathway is continually activated through the spontaneous hydrolysis of C3. Nonetheless, its activation is inherently maintained at a low level through the involvement of several complement regulatory proteins, with the most abundant among them being factor H [11]. The CFHR5 protein dimerizes into tissue-bound complement fragments and competitively controls the binding of factor H and C3b, activating the alternative pathway [12]. The P453S variant is located in SCR8, which is essential for the CFHR5 protein’s function.

In the current study, we have not directly demonstrated that the *P453S* variant inhibits factor H functionally, but multiple in silico analyses have demonstrated its pathogenic nature. Because we did not conduct genetic testing on the parents due to lack of consent, we couldn’t determine whether this case was sporadic or familial. The reported penetrance of the genetic abnormality associated with CFHR5 nephropathy on the island of Cyprus exceeds 90% [6]. However, there is a dearth of reported penetrance for other mutations, to the best of our knowledge.

Within the subset of C3 nephropathy, patients with *CFHR5* mutations are known to have a favorable renal prognosis [13]. Consistent with previous reports, the renal function of our patient remains stable. Elevated levels of sC5b-9, the final product of complement, and non-elevated intermediate products Ba and C5a in our patient may indicate slow activation of the alternative pathway. We speculate that the P453S mutation potentially augments the binding of the CFHR5 protein to C3b, subsequently reinforcing the effect of competitive inhibition on factor H. This, in turn, could have contributed to a gradual activation of complement pathway and resulted in a stable renal function for our patient. Therefore, the P453S variant could potentially play a significant role in CFHR5 protein’s function and renal prognosis.

## Conclusions

The case of a young woman with C3 nephritis and a heterozygous rare variant, P453S, in *CFHR5, *is described in this report. The novel P453S mutation reported in this study is positioned within a domain essential for CFHR5 protein function, and its pathogenicity has been substantiated through multiple in silico analyses. The P453S mutation potentially enhances the binding between CFHR5 protein and C3b, intensifying competitive inhibitory effects against H factor. This mutation could play a significant role in the function of CFHR5 protein and the prognosis of the kidneys. Accumulation of further variant reports is awaited to elucidate the pathogenesis of C3 nephritis.
